# Exercise improved lipid metabolism and insulin sensitivity in rats fed a high-fat diet by regulating glucose transporter 4 (GLUT4) and musclin expression

**DOI:** 10.1590/1414-431X20165129

**Published:** 2016-04-29

**Authors:** J. Yu, J. Zheng, X.F. Liu, Z.L. Feng, X.P. Zhang, L.L. Cao, Z.P. Zhou

**Affiliations:** Department of Endocrinology, The First Hospital of Jiujiang City, Jiangxi Province, China Jiujiang Affiliated Hospital of Nanchang University, Jiujiang, Jiangxi Province, China

**Keywords:** Insulin resistance, Musclin, GLUT4, Swimming, Triglyceride deposition

## Abstract

This study aimed to evaluate the effects of exercise training on triglyceride deposition and the expression of musclin and glucose transporter 4 (GLUT4) in a rat model of insulin resistance. Thirty male Sprague-Dawley rats (8 weeks old, weight 160±10 g) were fed a high-fat diet (40% calories from fat) and randomly divided into high-fat control group and swimming intervention group. Rats fed with standard food served as normal control. We found that 8-week swimming intervention significantly decreased body weight (from 516.23±46.27 to 455.43±32.55 g) and visceral fat content (from 39.36±2.50 to 33.02±2.24 g) but increased insulin sensitivity index of the rats fed with a high-fat diet. Moreover, swimming intervention improved serum levels of TG (from 1.40±0.83 to 0.58±0.26 mmol/L) and free fatty acids (from 837.80±164.25 to 556.38±144.77 μEq/L) as well as muscle triglycerides deposition (from 0.55±0.06 to 0.45±0.02 mmol/g) in rats fed a high-fat diet. Compared with rats fed a standard food, musclin expression was significantly elevated, while GLUT4 expression was decreased in the muscles of rats fed a high-fat diet. In sharp contrast, swimming intervention significantly reduced the expression of musclin and increased the expression of GLUT4 in the muscles of rats fed a high-fat diet. In conclusion, increased musclin expression may be associated with insulin resistance in skeletal muscle, and exercise training improves lipid metabolism and insulin sensitivity probably by upregulating GLUT4 and downregulating musclin.

## Introduction

Insulin resistance (IR) and functional impairment of islet β cells are the major pathological causes and hallmarks of type 2 diabetes. IR occurs prior to islet β cell damage, and is the common physiopathological basis for high blood pressure, high blood cholesterol, obesity, and cardiovascular disease (1). IR plays a pivotal role in the pathogenesis of type 2 diabetes, but the precise molecular mechanism remains largely unknown.

IR is a reduction of the responses of insulin target cells and tissues to a physiological concentration of insulin. IR is characterized by reduced insulin sensitivity of peripheral target tissues including muscle, lipid tissue and liver, and reduced metabolism of glucose. IR is known to cause metabolic disorders such as metabolic stress syndrome, obesity, high blood pressure, high blood lipids, high blood uric acid, and diabetes. Multiple factors contribute to IR. It is widely accepted that obesity, reduced physical activity and genetic alterations are the main risk factors for IR. During excessive caloric ingestion, plasma free fatty acids (FFAs) are deposited as triglycerides (TG) in non-fat cells, such as the muscle, liver, heart and pancreas. Accumulated deposition of FFAs in islet β cells leads to apoptosis of these cells, resulting in type 2 diabetes (2). Moreover, long-term high-fat or high-sugar diet can induce insulin resistance (3). Therefore, reducing FFAs is important for the prevention and treatment of insulin resistance.

Skeletal muscle participates in the metabolism of both glucose and fat, and plays an important role in maintaining the homeostasis of these two major processes. Skeletal muscle is one of the main target tissues of insulin and accounts for the metabolism of 80% of body glucose. Thus, it is important to elucidate the mechanism of insulin resistance in skeletal muscle. Musclin is a specific cytokine secreted by muscle cells. Musclin mRNA is almost exclusively expressed in muscle cells and its expression is significantly upregulated in obesity-induced insulin-resistant mice (4). Moreover, improvement of insulin resistance is accompanied by the upregulation of musclin expression (4). In addition, glucose transporter 4 (GLUT4), the peripheral tissue transporter of glucose, is mainly expressed in skeletal muscle and fat tissues, and is downregulated in diabetes (5,6). These facts suggest that the deregulation of musclin and GLUT4 plays an important role in insulin resistance of skeletal muscle.

Physical activity has been shown to significantly improve insulin sensitivity, presenting as an effective treatment strategy for insulin resistance. Physical activity increases the activity of AMP-dependent protein kinase (AMPK), thereby stimulating the uptake and metabolism of glucose and lipids in muscle (7). However, it is unknown whether physical activity improves insulin sensitivity by altering the expression of musclin. We hypothesized that exercise may improve lipid metabolism and insulin sensitivity by regulating musclin expression. In the present study, we aimed to evaluate the effects of exercise training on triglyceride deposition and the expression of musclin and GLUT4 using a rat model.

## Material and Methods

### Animals

Thirty male Sprague-Dawley rats (8 weeks old, weight 160±10 g) were obtained from Sino British SIPPR/BK Lab Animal (Shanghai, China). The protocol for animal experiments was approved by the Ethics Committee of The First People's Hospital of Jiujiang City, Jiugiang, China (#JJ04538). Rats were housed individually in polycarbonate cages and in controlled temperature (23±3°C) and humidity (60±5%), with a 12-h light/dark cycle. After acclimating to the environment for 1 week, the rats were randomly divided into the following 3 groups: normal control group (NC group), high-fat group (HF group), and exercise intervention group (SW group). The control group was fed a standard diet and the other two groups received a high-fat diet (40% calories from fat; Anlimo Technology, China) (8). After 16 weeks, rats in the SW group underwent improved Ploug swimming protocol for 8 weeks, as described previously (9). The rats swam for 30 min each day for the first week, and then for 1 h each day for the following 7 weeks to a total of 8 weeks, in a round stainless steel water tank of 70 dm^3^ volume and 50 cm depth, with water at 28±1°C. Five rats in each group were randomly selected to receive hyperinsulinemic-euglycemic clamp procedure (10). Meanwhile, blood glucose and body weight were monitored and recorded periodically. At the end of the 8-week exercise training, fasting blood was collected from *vena caudalis* for biochemical analysis. The rats were then weighed and sacrificed. Under sterile conditions, perirenal fat and epididymal adipose tissues were dissected, weighed, and calculated as visceral fat. The weight ratio of visceral fat/total weight of rats was calculated. The hindlimb gastrocnemius muscle was removed, washed with ice-cold normal saline and stored at -80°C for further analysis.

### Hyperinsulinemic-euglycemic clamp experiments

Hyperinsulinemic-euglycemic clamp experiment was performed as previously described (10). In brief, after fasting for 12 h, 5 rats in each group were anesthetized intraperitoneally (*ip*) with 1% pentobarbital sodium (wt/wt), followed by catheterization of the left carotid artery and internal jugular vein on both sides. Both sides of the jugular vein catheter were injected with saline, while the left carotid artery catheter was injected with heparin. After 30 min, 1 mL carotid artery blood was collected for the measurement of instant blood glucose with strip-operated blood glucose sensor (Accuchek; Roche, Germany), and basal insulin level. The left jugular vein catheter was connected to a syringe containing human insulin (Novolin R; Novo Nordisk, China), while the right jugular vein catheter was linked to infusion bags containing 10% glucose solution. The syringes and infusion bags were then connected to the two syringe pumps (Syringe pump; Terumo Holding; Japan) to make a continuous intravenous infusion. After continuous insulin infusion at a rate of 1.67 mU·kg^-1^·min^-1^ for 5 min, the instant blood glucose from the carotid artery was measured with strip-operated blood glucose sensor. When the values were above basal values within ±0.5 mmol/L, infusion of glucose began at a rate of 4-6 mg·kg^-1^·min^-1^. Blood glucose was measured every 5 min. When three continuous glucose values were within that range, insulin levels were measured again. During the whole procedure, blood glucose was measured 24 times. At the end, coefficient of variation of blood glucose (CVBG) was calculated.

### Measurement of insulin, serum FFA and TG

Serum FFA and TG were measured by an automatic biochemical analyzer (Olympus, Japan), while plasma insulin was measured by radioimmunoassay kit from North Biotechnology Research Institution (Beijing, China) according to the manufacturer's instructions.

### Determination of muscle triglyceride deposition

Muscle TG deposition was determined as described (11). Briefly, 100 mg of mixed muscle fiber from the hindlimb gastrocnemius muscle was added to 1.5 mL ethanol-acetone (1:1) solution, and ground to homogenize. Following centrifugation at 1,000 *g* for 15 min, the supernatant was collected for the measurement of TG on a WD21E Semi-automatic Biochemical Analyzer (Kangjin Medical, China) according to the manufacturer's instructions.

### Real-time-PCR

Total RNA was extracted from a mixture of 100 mg of muscle fiber from the hindlimb gastrocnemius muscle with Trizol reagent (USA). Reverse transcription was performed on 2 μg of total RNA using a High Capacity cDNA Reverse Transcription Kit from Tiangen Biotech (China) according to the manufacturer's instructions. Real-time PCR was performed on the 7900HT Fast Real-Time PCR System using the TaqMan^¯^ Universal Mastermix II (Applied Biosystems, USA). Rat musclin and GLUT4 expression was quantified with musclin and GLUT4 specific FAM™ dye-labeled MGB-probes (SBS Genetech, China) and normalized to β-actin (Applied Biosystems). The sequences of the primers were as follows: musclin, forward 5'-ACACTTCCTCCTGGCTAT-3′, reverse 5′-GAAGCATTTGCGGTGGACGAT-3′; GLUT4, forward 5′-GCCTTCTTTGAGATTGGTCC-3′, reverse 5′-CTGCTGTTTCCTTCATCCTG-3′; β-actin, forward 5′-CTTGGGTATGGAATCCTGTGG-3′, reverse 5′-CGGACTCATCGTACTCCTGCTT-3′.

### Western blot analysis

The muscle tissues were lysed on ice in RIPA lysis buffer (Cell Signaling Technology, USA) supplemented with protease inhibitors and phosphatase inhibitors (Roche, USA), and 1 mM PMSF (Sigma, USA). Equal amount of proteins were separated on 10% SDS-PAGE gels and transferred onto nitrocellulose membranes (Bio-Rad, USA), which were subsequently blocked with 5% skimmed milk. The membranes were incubated with primary antibodies against musclin (Santa Cruz Biotechnology, USA), GLUT4 and β-actin (Sigma). After washes with 0.1% Tween 20 in TBS, the membranes were incubated with horseradish peroxidase-conjugated secondary antibody (anti-mouse or anti-goat IgG, Sigma) and bound antibody was detected by enhanced chemiluminescence (Roche).

### Statistical analysis

Data are reported as means±SE. Statistical analysis was conducted using GraphPad Prism 5.0 software (GraphPad Software Inc., USA). Comparisons among multiple groups were performed using one-way ANOVA followed by a Newman-Keuls test. P<0.05 was considered to be statistically significant.

## Results

### Exercise intervention significantly alleviated high-fat diet-induced increase of body weight in rats

**Figure 1:**
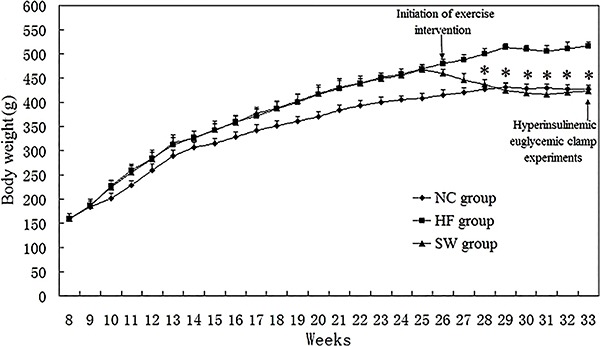
Alteration of body weight in rats from normal control group (NC group), high-fat diet group (HF group) and exercise intervention group (SW group). Food intake and body weight were monitored weekly. Rats of the SW group at 26 weeks of age were subjected to the improved Ploug swimming protocol for 8 weeks. At the end of the experiment, 5 rats in each group were randomly selected for hyperinsulinemic-euglycemic clamp analysis. Data are reported as means±SE of 10 animals. *P<0.05, HF group *vs* SW group (ANOVA followed by a Newman-Keuls test).

Compared with the NC group, body weight was significantly higher in the HF group (Figure 1, 475.36±37.32 *vs* 516.23±46.27 g). However, after the exercise intervention, body weight of the SW group was lower compared to the HF group (455.43±32.55 *vs* 516.23±46.27 g; P<0.05). Meanwhile, the body weight of the SW group dropped, week by week, from the age of 26 to 29 weeks. Moreover, there was no significant difference in the body weight between the SW group and the NC group from the age of 29 to 33 weeks.

### Exercise intervention improved the disposal of visceral fat, FFA and TG in rats fed a high-fat diet

To assess the effect of exercise intervention on lipid metabolism of obese rats, the visceral fat, FFA and TG of rats were measured. As shown in Table 1, the body weight, visceral fat, FFA, TG, and visceral fat/body weight ratio in the HF group were significantly higher than in the rats of the NC and SW groups. However, there were no significant differences in the body weight, visceral fat, FFA, TG, and visceral fat/body weight ratio between the NC group and the SW group.

**Table:**
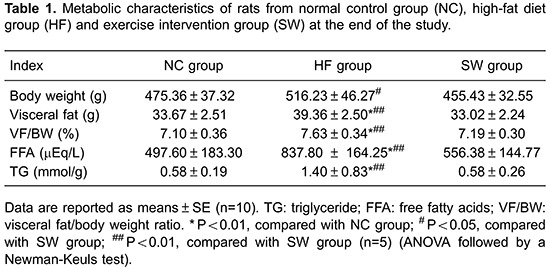

### Exercise intervention improved systemic insulin sensitivity of rats fed with high-fat diet

We performed hyperinsulinemic-euglycemic clamp experiments and found that both body weight and basic serum insulin level were significantly higher in the HF group than in either the NC group or the SW group, but there were no significant differences in basic blood glucose, steady-state blood glucose and steady-state serum insulin levels among the three groups (Table 2). In addition, compared to the HF group, rats in the NC and SW groups required 23% higher glucose infusion rate in order to maintain euglycemia, indicating reduced insulin sensitivity of rats in the HF group. Meanwhile, the average CVBG in these experiments was 7.56%, reflecting the reliability of the clamp experiments.

**Table:**
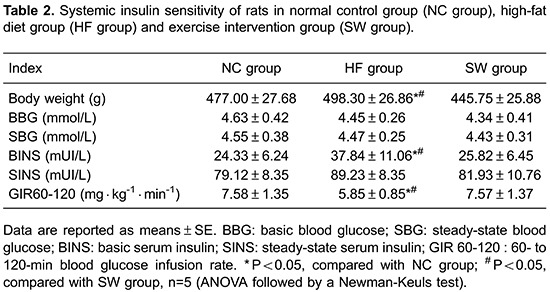

### Exercise intervention improved muscle TG deposition

**Figure 2:**
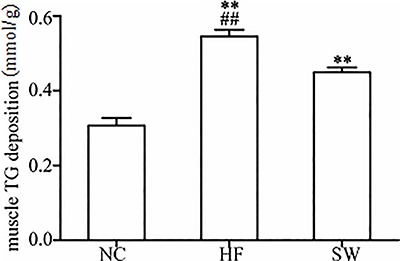
Effects of exercise intervention on muscle triglycerides (TG) deposition in isolated hindlimb gastrocnemius muscle of rats from normal control group (NC group), high-fat diet group (HF group) and exercise intervention group (SW group). Data are reported as means±SE (n=10). **P<0.01 *vs* NC group; ^# #^ P<0.01 *vs* SW group (ANOVA followed by a Newman-Keuls test).

To investigate the effect of exercise intervention on TG metabolism in the muscle of obese rats, we examined TG deposition in isolated hindlimb gastrocnemius muscle from 33-week old rats. The results showed that skeletal muscle TG deposition in rats fed a high-fat diet was significantly higher (0.55±0.06 mmol/g) than that of either the NC group (0.32±0.07 mmol/g) or the SW group (0.45±0.02 mmol/g) (P<0.01; Figure 2). Meanwhile, we found that skeletal muscle TG deposition in the SW group was significantly higher than that of the NC group (P<0.01; Figure 2).

### Exercise intervention increased the expression of GLUT4 and decreased the expression of musclin in rats fed a high-fat diet

**Figure 3:**
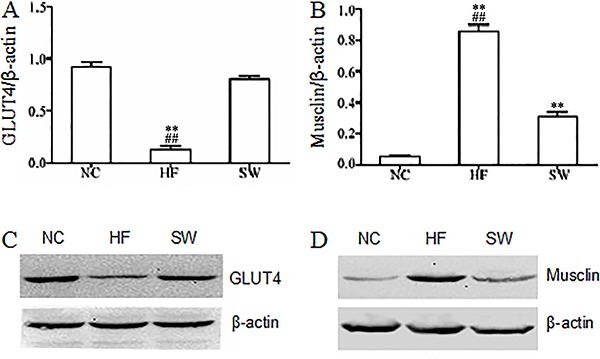
Effects of exercise intervention on the expression of GLUT4 and musclin in isolated hindlimb gastrocnemius muscle of rats from normal control group (NC group), high-fat diet group (HF group) and exercise intervention group (SW group). RT-PCR analysis of relative GLUT4 mRNA level (*A*) and relative musclin mRNA level (*B*) with β-actin as internal control. Western blot analysis of GLUT4 protein level (*C*) and musclin protein level (*D*) with β-actin as loading control. Data are reported as means±SE (n=10). **P<0.01 *vs* NC group; ^# #^P<0.01 *vs* SW group (ANOVA followed by a Newman-Keuls test).

To investigate the underlying molecular mechanism by which exercise training improves insulin sensitivity in fat rats, we determined the mRNA and protein expression levels of GLUT4 and musclin in skeletal muscle from the three groups. As shown in Figure 3A, the expression of GLUT4 mRNA was significantly lower in the HF group compared to the NC group. In contrast, rats in the SW group displayed significantly higher GLUT4 mRNA levels compared to the HF group (P<0.01). Musclin mRNA levels in the HF group was 6.5 and 2.6 times higher than that of the NC and SW groups, respectively (P<0.01), while musclin mRNA level was significantly higher in the SW group than in the NC group (P<0.01; Figure 3B). Western blot analysis demonstrates that GLUT4 protein level was significantly lower in the HF group compared to the NC group (P<0.01), while GLUT4 protein level was significantly higher in the SW group compared to the HF group (P<0.01; Figure 3C). Similar to mRNA level, musclin protein level in the HF group was significantly higher compared to the NC and SW groups (P<0.01). Moreover, musclin protein level in the SW group was significantly higher than in the NC group (P<0.01; Figure 3D).

## Discussion

In this study, we established insulin resistance in rats by feeding a high-fat diet, which demonstrated higher body weight, serum insulin level, visceral fat, FFAs, visceral fat/body weight ratio, and skeletal muscle TG deposition, all of which were significantly attenuated by exercise intervention. Most importantly, we found that exercise training significantly increased the expression of GLUT4 and decreased the expression of musclin in insulin-resistant rats fed a high-fat diet.

It has been well established that lipid metabolism abnormalities are closely associated with insulin resistance. Increased visceral fat is an important risk factor for insulin resistance (12). Accumulation of visceral fat leads to increased release of FFAs and liver TG synthesis, resulting in lipids and glucose metabolism disorders ([Bibr B13] [Bibr B14] 1315). Moreover, elevation of FFAs inhibits the intake and utilization of muscle glucose through activation of protein kinase C, and suppresses the activity of the insulin receptor signaling pathway, leading to insulin resistance ([Bibr B16] [Bibr B17]-18). In this study, we found that a long period of high-fat feeding resulted in remarkable insulin resistance as demonstrated by the elevation of blood sugar, blood lipids (TG, FFAs), serum insulin and visceral fat, providing additional evidence for the ‘lipid toxicity' proposal, that stipulates lipid metabolism abnormality as the key etiological event for type 2 diabetes (19).

Musclin plays a key role in skeletal muscle, maintaining the homeostasis of sugar and lipid metabolism, but the underlying mechanism remains elusive. It was reported that musclin mRNA was detectable only in skeletal muscle of both mouse and rat (4). The mRNA expression of musclin is controlled by nutritional status and hormone factors, especially insulin (20). In this study, we found that both the mRNA and protein levels of musclin were significantly increased in insulin-resistant rats, suggesting an important role for musclin in insulin resistance of skeletal muscle. GLUT4 is a 509 amino acid transmembrane glycoprotein and the main glucose transporter in insulin sensitive skeletal muscle, myocardial and fat tissues (21). High fat diet decreases the expression of GLUT4 and hence, inhibits glucose uptake by skeletal muscle, leading to insulin resistance (22). Consistent with these previous observations, our results show decreased GLUT4 expression at both mRNA and protein levels in insulin-resistant rats fed a high-fat diet.

Amounting data have shown that physical activity is an effective approach for improving insulin resistance by increasing the metabolism of glucose and lipids (17). Furthermore, swimming has been shown to improve muscle insulin sensitivity in high-fat diet-induced obese rats (23,24). In this study, swimming intervention increased glucose infusion rate, blood TG and FFA, and muscle TG deposition compared to control groups. In addition, swimming intervention significantly reduced the expression of musclin while increased the expression of GLUT4. Our results suggest that swimming may attenuate insulin resistance by altering the expression of musclin and GLUT4. A very recent study suggested that musclin is an important exercise-stimulated myokine that enhances physical endurance and metabolic well-being (25). It will be of importance to elucidate the molecular mechanism by which exercise training changes the expression of musclin and GLUT4 in further investigations.

In conclusion, the long-term high-fat diet led to an increase of body weight, serum insulin level, visceral fat, FFA, visceral fat/body weight ratio, and skeletal muscle TG deposition, all of which can be attenuated by exercise training. Moreover, increased expression of musclin and decreased expression of GLUT4 in insulin-resistant obese rats could be abolished by exercise training. Our data suggest that increased musclin expression may be associated with insulin resistance in skeletal muscle, and exercise training improved lipid metabolism and insulin sensitivity, probably by upregulating GLUT4 and downregulating musclin.
